# Novel Pyrazolo[3,4-*d*]pyrimidine Derivatives as Potential Antitumor Agents: Exploratory Synthesis, Preliminary Structure-Activity Relationships, and *in Vitro* Biological Evaluation

**DOI:** 10.3390/molecules161210685

**Published:** 2011-12-20

**Authors:** Hai-Yun He, Jin-Ni Zhao, Ruo Jia, Ying-Lan Zhao, Sheng-Yong Yang, Luo-Ting Yu, Li Yang

**Affiliations:** 1 State Key Laboratory of Biotherapy and Cancer Center, West China Hospital, West China Medicinal School, Sichuan University, Chengdu 610041, Sichuan, China; 2 College of Chemistry and Chemical Engineering, Chongqing University of Science and Technology, Chongqing 401331, China

**Keywords:** pyrazolo[3,4-*d*]pyrimidine, exploratory synthesis, anticancer activity, apoptosis

## Abstract

In a cell-based screen of novel anticancer agents, the hit compound **1a** which bears a pyrazolo[3,4-*d*]pyrimidine scaffold exhibited high inhibitory activity against a panel of four different types of tumor cell lines. In particular, the IC_50_ for A549 cells was 2.24 µM, compared with an IC_50_ of 9.20 µM for doxorubicin, the positive control. Four synthetic routes of the key intermediate **3** of **1a** were explored and **1a** was prepared via route D on the gram scale for further research. Two analogs of **1a** were synthesized and their preliminary structure-activity relationships were studied. Flow cytometric analysis revealed that compound **1a** could significantly induce apoptosis in A549 cells *in vitro* at low micromolar concentrations. These results suggest that the target compound **1a** and its analogs with the pyrazolo[3,4-*d*]pyrimidine scaffold might potentially constitute a novel class of anticancer agents, which requires further studies.

## 1. Introduction

Cancer is a major health problem worldwide. Improvements in treatment and prevention have led to a decrease in cancer deaths, but the number of new diagnoses continues to rise [1]. Chemotherapy is one of the most commonly used treatment options, especially for unresectable patients. However, the use of conventional cytotoxic drugs, including doxorubicin, cisplatin and fluorouracil, has not shown any improvement in survival, and severe adverse effects have been frequently observed in treated patients [2]. Thus, it is urgent to develop novel chemotherapeutic agents for the treatment of cancer.

**Figure 1:**
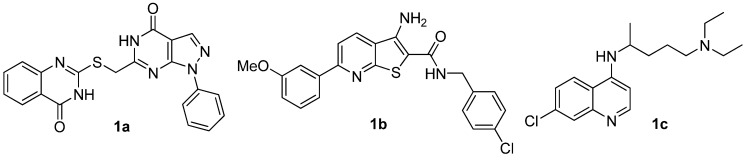
The hit compounds in the cell-based screening of anticancer agents.

Our research group has been interested in the design, screening, synthesis and biological evaluation of novel tumor growth inhibitors and apoptosis inducers as potential anticancer agents [3,4,5,6,7]. Recently, a series of promising hit compounds, such as **1a** [8], **1b** [9] and **1c** [10], *etc.* (Figure 1) have been found in a cell-based screen of anticancer agents. Among them, compound **1a** with a pyrazolo[3,4-*d*]- pyrimidine scaffold showed broad-spectrum anticancer activity *in vitro*. Low micromolar inhibition potency was demonstrated against several tumor cell lines including A549, MCF-7, HepG2 and PC-3 using the MTT assay (Table 1). For example, the IC_50_ for the A549 cell line was 2.24 µM.

**Table 1:** The anti-proliferation activities of compounds **1a**, **1d** and **1e** against various cancer cell lines.

Compound	IC_50 _(µM) ^a^			
	HepG2	MCF-7	A549	PC-3
**1a**	13.9	42.3	**2.24**	26.6
**1d**	25.2	**1.74**	**5.20**	>100
**1e**	>100	>100	47.0	>100
Doxorubicin	0.54	0.75	9.20	0.60

^a^ The cytotoxicity effects of compounds on various cancer cell lines were determined by the MTT assay [30]. The results were expressed as the IC_50_, and were the means calculated from three independent experiments.

Pyrazolo[3,4-*d*]pyrimidine derivatives demonstrate various biological activities, such as inhibition of phosphodiesterase-5 (PDE5) [11], modulation of the human adenosine receptor [12], growth inhibition of Gram-positive (Gr^+^) bacteria [13], memory modulation [14], *etc.* To the best of our knowledge, although the antitumor activity of pyrazolo[3,4-*d*]pyrimidine derivatives has been reported [15,16,17], the antitumor activity of 2- methyl thioether**-**substituted pyrazolo[3,4-*d*]pyrimidine derivatives such as **1a** is being reported for the first time by our group [18]. Based on the promising *in vitro* activity of **1a** and its structural novelty, we examined its antitumor activity further. Herein, we report the exploratory synthesis of the key intermediate **3** of **1a**, the subsequent synthesis of **1a** together with its analogs, preliminary structure-activity relationships (SARs) and biological evaluation of this novel class of anticancer agents.

## 2. Results and Discussion

### 2.1. Chemistry

**Scheme 1:**
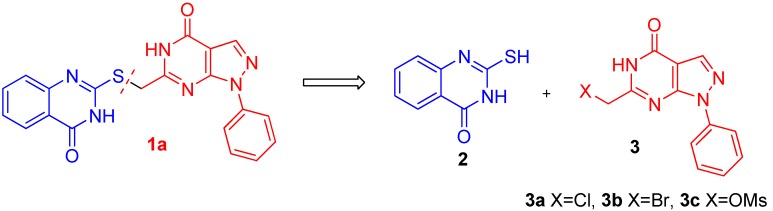
The retrosynthetic analysis of **1a**.

To the best of our knowledge, the synthesis of **1a** has not been previously reported [18]. Therefore, the synthetic route of **1a** had to be designed to provide enough sample to gain insight into the SAR followed by further studies examining its antitumor activity. According to the retrosynthetic analysis (Scheme 1), the target compound **1a** could be obtained through the condensation under basic conditions of **3** (**3a**, **3b** or **3c**) and **2**, which is commercially available. Thus, the key to synthesizing compound **1a** lay in the acquisition of the key intermediate **3**.

**Scheme 2:**
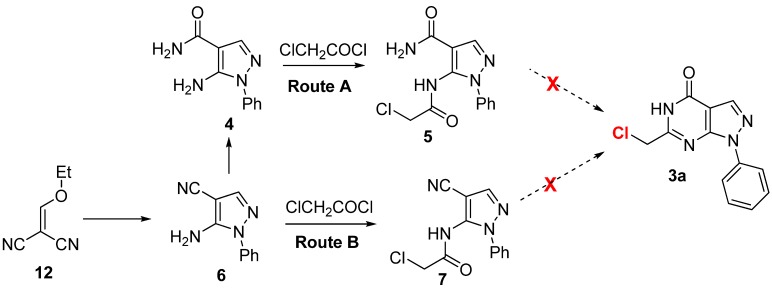
The synthetic routes A and B to **3a**.

The only synthesis report [19] available for the key intermediate compound **3a** was route A in Scheme 2, in which readily available compound **4** [20] reacted with 2-chloroacetyl chloride to yield acylated product **5**, which underwent subsequent ring closure under basic conditions to produce **3a**. However, we were unable to obtain the annulated product **3a** following this literature method. We then attempted to produce **3a** via route B in Scheme 2: Compound **6**, which is also the precursor of **4** was subjected to acylation and ring closure under oxidative conditions [21]. We were also unable to obtain **3a** by this method.

**Scheme 3:**
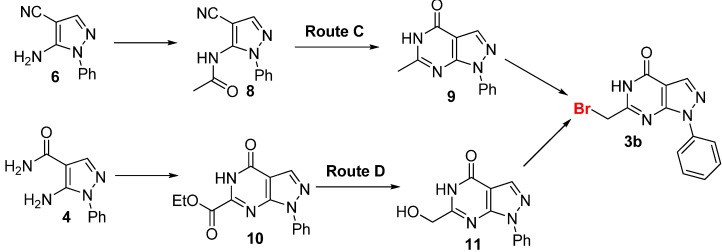
The synthetic route C and D of **3b**.

We concluded that the failure of of **5** or **7** to cyclize might result from the bulky phenyl group on the pyrazole ring. Thus, bromination of the methyl group of known compound **9** [22], which has a *N*-phenyl substituted pyrazolo[3,4-*d*]pyrimidine scaffold, was attempted in order to generate brominated product **3b** **(**Route C in Scheme 3**)**. Under mild bromination conditions (Br_2_/HAc at 60 °C) [23], we obtained **3b** with approximately 10% conversion of **9** according to the ^1^H-NMR and MS analysis of the crude product. The conversion of compound **9** into **3a** under harsher bromination conditions (Br_2_/HAc at 90 °C) was aborted due to the presence of unidentified impurities that were too difficult to remove to obtain the purified product.

**Scheme 4:**
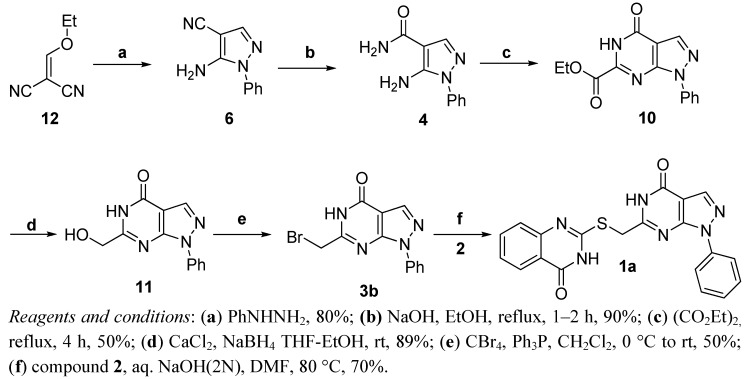
Synthetic route D to **1a**.

We then used route D in Scheme 3 to generate **3b**. A 60% yield of compound **3b** was obtained by reduction of **10** to give **11** [24] followed by substitution of the hydroxyl group by a Br atom with CBr_4_/Ph_3_P [25] to generate **3b**. Thus, the practical synthesis of target compound **1a** was achieved (Scheme 4).

As shown in Scheme 4, treatment of the commercially available starting material **12** with phenylhydrazine gave the pyrazole derivative **6** with a yield of 80%. Then, **6** was readily converted to its amide derivative **4** in refluxing ethanol in the presence of sodium hydroxide [26]. Treatment of **4** with neat diethyl oxalate under reflux [27] generated the annulated product **10** in 40% yield, followed by reduction with sodium borohydride and calcium chloride in THF-ethanol [28] to give **11**. The key intermediate **3b** could be then generated by bromination of **11** with carbon tetrabromide and triphenylphosphine. Finally, **3b** was condensed with **2** under the action of potassium carbonate in DMF to give **1a** with a yield of 76% on the gram scale. The structure of **1a** was characterized by ^1^H-NMR and ESI-MS analysis.

### 2.2. Anticancer Activity

**Figure 2:**
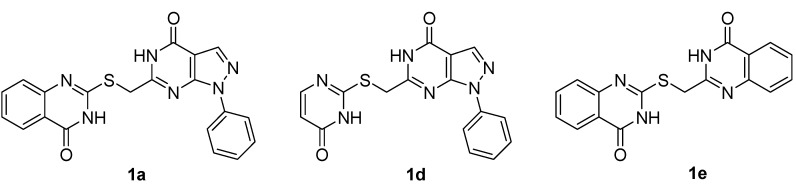
The structure of compound **1a** and its analogs **1d** and **1e**.

To provide insight into the SAR for further research, two analogs of **1a**, that is **1d** and **1e **(Figure 2), were synthesized following a similar synthetic route to that used for **1a** [29].

The antiproliferative activities of compounds **1a**, **1d** and **1e** were examined in a range of cancer cell lines including human hepatoma HepG2, breast cancer cell line MCF-7, lung cancer cell line A549, and prostate cancer cell line PC-3 using the MTT assay with doxorubicin as a positive control. The experimental data are summarized in Table 1.

As expected, the biological activity of **1e** in which the characteristic pyrazolo[3,4-*d*]pyrimidine scaffold was changed decreased significantly, suggesting that the pyrazolo[3,4-*d*]pyrimidine scaffold is pivotal to the anti-proliferative activity. It is noteworthy that the analog **1d** with the pyrazolo[3,4-*d*]- pyrimidine scaffold inhibited proliferation of the human breast MCF-7 cells with an IC_50_ value of 1.74 µM (compared with the parent compound **1a**, whose IC_50_ was 42.3 µM). These results suggest that the target compound **1a** and its analogs with the pyrazolo[3,4-*d*]pyrimidine scaffold might potentially constitute a novel class of anticancer agents, which require further studies.

**Figure 3:**
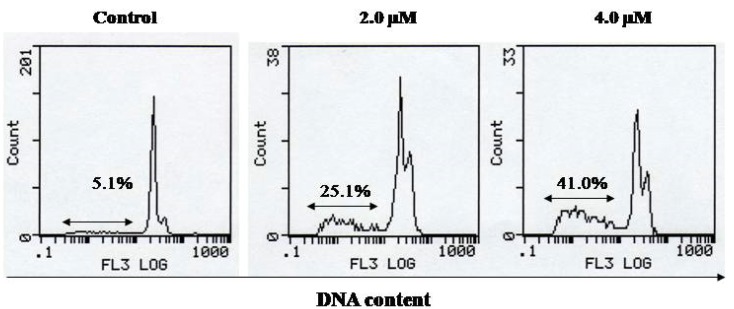
Effect of **1a** on the induction of apoptosis in A549 cells. Cells were treated with 2.0 µM and 4.0 µM of **1a** for 48 h. The cells in the sub-G_1_ phase were considered to be apoptotic cells (n = 5).

To further disclose the anti-proliferative mechanism of **1a**, flow cytometric analysis was used to identify and measure the apoptotic cells (sub-G_1_ cells) and the cell cycle after propidium iodide (PI) staining, as described previously [31]. The data are shown in Figure 3.

Exposure of A549 cells to compound **1a** (2.0–4.0 µM) for 48 h resulted in a distinct sub-G_1_ peak that represents the population of apoptotic cells [32]. The percentage of cells in the sub-G_1_ phase was 25.1%–41.0% for compound **1a** (2.0–4.0 µM) and 5.1% for the control. These results suggest that compound **1a** could significantly induce apoptosis in the lung cancer cell line A549 *in vitro* at low micromolar concentrations.

## 3. Experimental

### General

All solvents and reagents were analytical grade pure and used without further purification. ^1^H-NMR spectra were recorded on a Bruker Avance (Varian Unity Inova) 400 MHz spectrometer using TMS as internal reference chemical shift in δ, ppm. Low resolution ESI-MS spectra were carried out on a Waters triquadrupole mass spectrometer.

*5-Amino-1-phenyl-1H-pyrazole-4-carbonitrile* (**6**). To a solution of 2-(ethoxymethylene)malononitrile **12** (1.50 g, 12.3 mmol) in ethanol (10.0 mL) was added phenylhydrazine (2.66 g, 24.6 mmol) dropwise. After stirring at room temperature for 1 h and then under reflux for 2.5 h, the reaction mixture was poured into water, and the residue after concentration *in vacuo* was cooled overnight. The precipitate was collected by filtration, washed with water and dried to afford 1.81g of a yellow solid. Yield: 80%. ^1^H-NMR (DMSO-*d_6_*): δ 7.79 (s, 1H), 7.52–7.56 (m, 4H), 7.41–7.51 (m, 1H), 6.70 (s, 2H). ESI-MS: *m/z* 183.11[M-H]^+^.

*5-Amino-1-phenyl-1H-pyrazole-4-carboxamide* (**4**). To a solution of **6** (1.22 g, 11.3 mmol) in ethanol (10.0 mL) was added dropwise a solution of 33.3% sodium hydroxide (12.0 mL, 24.6 mmol). The mixture was then heated under reflux for 5 h. After cooling to room temperature, the reaction mixture was concentrated under vacuum and 6.00 mol/L HCl solution were added until pH 4. After cooling in ice water for 4 h, the precipitated solid was filtered, washed with water and dried to give 1.21 g of white product (90% yield).^1^H-NMR (DMSO-*d_6_*): δ 8.32 (s, 1H), 7.50–7.90 (m, 4H), 7.32–7.40 (m, 1H), 6.85 (s, 2H), 6.37 (s, 2H).ESI-MS: *m/z* 201.16 [M-H]^+^.

*Ethyl 4-oxo-1-phenyl-4,5-dihydro-1H-pyrazolo[3,4-d]pyrimidine-6-carboxylate* (**10**). A mixture of **4** (2.50 g, 12.40 mmol) and diethyl oxalate (8.2 mL) was refluxed for 5 h and the reaction mixture was allowed to cool to room temperature overnight. The precipitate was collected, washed with petroleum ether and dried to give 1.75 g of a white solid. Yield: 50%. ^1^H-NMR (DMSO-*d_6_*): δ 12.87 (s, 1H), 8.44 (s, 1H), 8.03 (d, *J* = 8.0 Hz, 2H), 7.60 (t, *J* = 7.8 Hz, 2H), 7.45 (t, *J* = 7.8 Hz, 1H), 4.39 (q, *J* = 7.2 Hz, 2H), 1.35 (t, *J* = 7.2 Hz, 3H). ESI-MS: *m/z* 283.20 [M-H]^+^.

*6-(Hydroxymethyl)-1-phenyl-1H-pyrazolo[3,4-d]pyrimidin-4(5-H)-one* (**11**). To a solution of **10** (2.29 g, 8.06 mmol) in THF/H_2_O (170 mL/55 mL) was added calcium chloride anhydrous (1.78 g, 16.10 mmol) and sodium borohydride (1.23 g, 32.2 mmol), the resulting mixture was stirred at room temperature overnight. The reaction mixture was poured in ethyl acetate (150 mL) and the organic phase separated was washed twice with brine (50 mL) and then dried over sodium sulfate. The residue after evaporating *in vacuo* was recrystallized from ethanol to afford 1.74 g of a solid. Yield: 89%. ^1^H-NMR (DMSO-*d_6_*): δ 12.04 (s, 1H), 8.30 (s, 1H), 8.06–8.08 (d, *J* = 8.8 Hz, 2H), 7.54–7.59 (t, *J* = 8.8 Hz, 2H), 7.37–7.42 (t, *J* = 1.2 Hz, 1H), 5.74 (s, br, 1H,), 4.45 (s, 2H). ESI-MS: *m/z* 241.15 [M-H]^+^.

*6-(Bromomethyl)-1-phenyl-1H-pyrazolo[3,4-d]pyrimidin-4(5H)-one* (**3b**). **11** (166 mg, 1.0 mmol) and carbon tetrabromide (331.6 mg, 2.00 mmol) were dissolved in dichloromethane (5.0 mL), a solution of triphenyl phosohine (656 mg, 2.50 mmol) in dichloromethane (1.5 mL) was dropped in at 0 °C. After stirring at room temperature for 3 h, the reaction mixture were concentrated *in vacuo* and the residue is chromatographed on silica gel using ethyl acetate/petroleum ether (1/4 by volume) as eluent to afford 63.0 mg of a white solid. Yield: 50%. ^1^H-NMR (DMSO-*d_6_*): δ 12.76 (s, 1H), 8.35 (s, 1H), 8.02–8.05 (m, 2H), 7.55–7.62 (m, 2H), 7.40–7.44 (m, 1H), 4.47 (s, 2H). ESI-MS: *m/z* 305.29 [M-H]^+^.

*2-((4-Oxo-1-phenyl-4,5-dihydro-1H-pyrazolo[3,4-d]pyrimidin-6-yl)methylthio)quinazolin-4(3H)-one* (**1a**). To a solution of ethyl 2-mercaptoquinazolin-4(3*H*)-one (**2**) (34.5 mg, 0.21 mmol) in ethanol (2.5 mL) was added 2 mol/L solution of NaOH (0.2 mL), the temperature was raised to 50 °C. And (**3b**) (62.5 mg, 0.21 mmol) was added. About 3 h later, the reaction mixture was cooled and poured into a solution of 2 mol/L NaHSO_4_ (2.0 mL). The precipitated solid was collected and crystallized to give 57.6 mg of a green solid. Yield: 70%. ^1^H-NMR (DMSO-*d_6_*): δ 12.80 (s, 1H), 12.73 (s, 1H), 8.36 (s, 1H), 8.05–8.11 (m, 3H), 7.82–7.86 (m, 1H), 7.38–7.59 (m, 5H), 4.63 (s, 2H). ESI-MS: *m/z* 401.00 [M-H]^+^.

*6-((6-Oxo-1,6-dihydropyrimidin-2-ylthio)methyl)-1-phenyl-1H-pyrazolo[3,4-d]pyrimidin-4(5H)-one* (**1d**). To a solution of 2-mercaptopyrimidin-4(*3H*)-one (76.9 mg, 0.60 mmol) in ethanol (3.0 mL) was added 2 mol/L solution of NaOH (0.3 mL), the temperature was raised to 60 °C and then **3b** (183.0 mg, 0.60 mmol) was added. About 10 h later, the reaction mixture was cooled and NaHSO_4_ (0.06 g) was added followed by agitation. The precipitated solid was collected and chromatographed on silica gel with ethyl acetate/petroleum ether (1/4 by volume) as eluent to afford 93.8 mg of a green solid. Yield: 53%. ^1^H-NMR (DMSO-*d_6_*): δ 11.00–14.12 (br, 2H), δ 8.30 (s, 1H), 8.05 (d, *J* = 7.6 Hz, 2H), 7.75 (d, *J* = 6.4 Hz, 1H), 7.53 (t, *J* = 7.6 Hz, 2H), 7.39 (d, *J* = 7.6 Hz, 1H), 5.94 (d, *J* = 6.4 Hz, 1H), 4.32 (s, 2H). ESI-MS: *m/z* 353.07 [M-H]^+^.

*2-((4-Oxo-3,4-dihydroquinazolin-2-yl)methylthio)quinazolin-4(3H)-one* (**1e**). To a solution of 2-mercaptopyrimidin-4(*3H*)-one (213.9 mg, 1.11 mmol) in ethanol (6.0 mL) was added a 2 mol/L solution of NaOH (1.0 mL), the temperature was raised to 50 °C and 2-chloroquinazolin-4(*3H*)-one (194.6 mg, 1.08 mmol) was added to the resulting mixture. After stirring overnight, the reaction mixture was cooled and NaHSO_4_ (0.12 g) was added. The precipitated solid was collected and and chromatographed on silica gel with ethyl acetate/petroleum ether (1/4 by volume) as eluent to afford 299.5 mg of a white solid. Yield: 83%. ^1^H-NMR (DMSO-*d_6_*): δ 12.70 (s, br, 2H), 8.10 (d, *J* = 8.0 Hz, 1H), 8.03 (d, *J* = 7.6 Hz, 1H), 7.77 (q, *J* = 7.2 Hz, 2H), 7.62 (d, *J* = 8.4 Hz, 1H), 7.50 (t, *J* = 9.2 Hz, 2H), 7.40 (t, *J* = 7.6 Hz, 1H). ESI-MS: *m/z* 337.17 [M-H]^+^.

## 4. Conclusions

In conclusion, four synthetic routes to **3a**, which is the key intermediate of a promising target compound **1a** found in a cell-based screen of anticancer agents, were examined. Based on these findings, **1a** was prepared on the gram scale for further research. Compound **1a** exhibited high anticancer activity *in vitro*, especially for the lung cancer cell line A549 (the IC_50_ for A549 cells was 2.24 µM). Flow cytometric analysis of compound **1a** revealed that it could significantly induce apoptosis in A549 cells *in vitro* at low micromolar concentrations. Moreover, two analogs of **1a** were synthesized and their *in vitro* anticancer activities were tested. Further SAR and mechanism studies are currently in progress.
